# Renin–angiotensin system dysregulation in critically ill patients with acute respiratory distress syndrome due to COVID-19: a preliminary report

**DOI:** 10.1186/s13054-021-03507-7

**Published:** 2021-03-01

**Authors:** Davide Eleuteri, Luca Montini, Salvatore Lucio Cutuli, Cristina Rossi, Francesca Alcaro, Massimo Antonelli

**Affiliations:** 1 Department of Intensive Care Medicine and Anesthesiology, Fondazione Policlinico Universitario Agostino Gemelli IRCCS, Rome, Italy; 2 Università Cattolica del Sacro Cuore, Rome, Italy; 3 Department of Laboratory Diagnostic and Infectious Diseases, Fondazione Policlinico Universitario Agostino Gemelli IRCCS, Rome, Italy

The renin–angiotensin system (RAS) may have an important role into the pathogenesis of the COVID-19. Indeed, SARS-CoV-2 binds to angiotensin-converting enzyme 2 (ACE2) to gain entry to host cells. ACE2 is a peptidase that cleaves the potent vasoconstrictor angiotensin II (Ang II) to generate angiotensin 1–7 (Ang 1–7), a heptapeptide having vasodilator and anti-inflammatory function. Thus, ACE2 is a crucial counter-regulatory component of the RAS [1]. From March 9 to March 27, 2020, we analyzed plasma levels of renin, angiotensin I (Ang I), Ang II and Ang 1–7 in 32 consecutive patients with laboratory-confirmed SARS-Cov-2 infection and acute respiratory distress syndrome (ARDS) within 24 h of admission to ICU, approved by the Ethical Committee of Fondazione Policlinico Gemelli. All samples were drawn at morning. Before sample collection the hemodynamic parameters were optimized according to standard of care of our institution. RAS peptide concentrations were compared between survivors and non-survivors and correlated with clinical parameters. Compared to survivors, non-survivors had higher serum renin with lower Ang I, Ang II and Ang 1–7 (Table 1). The results were similar excluding from analysis 4 patients who were taking RAS inhibitors at inclusion (Table 2). Patients who required invasive mechanical ventilation (IMV) had lower Ang 1–7 than patients who never required IMV (214 pg/ml [IQR: 163–298] vs 335 [IQR: 259–499], *p* = 0.01). Our observations are consistent with a previous study on patients with vasodilatory shock, demonstrating that renin levels above the median of study population were associated with an increased risk of mortality. In these patients, treatment with synthetic Ang II reduced renin concentrations and the risk of mortality [2]. Therefore, the authors speculated that exogenous Ang II modulated the inflammatory response caused by excess renin. Furthermore, renin concentrations were positively correlated to Ang I/II ratios denoting an impaired conversion of Ang I to Ang II by angiotensin-converting enzyme (ACE). In our research, although not correlated to serum renin, Ang I/II ratios were markedly higher than those reported in healthy subjects (median Ang I/II ratio 1.8 vs 0.4, respectively) [3]. This relative Ang II deficiency is coherent with older studies on patients with ARDS unrelated to SARS-CoV-2 reporting a defect of endothelial–bound ACE activity due to endothelial injury [4]. Indeed, in patients with COVID-19 there is evidence of vascular involvement with diffuse inflammation which can result in widespread endothelial dysfunction [3, 5]. However, we did not measure all the angiotensin peptides, and hence, we cannot exclude an enhanced conversion of Ang I and Ang II to downstream products other than Ang 1–7, such as angiotensin 1–9 and angiotensin 1–5. Previous studies reported that some patients with COVID-19 and acute hypoxemic respiratory failure present near normal lung mechanics indicating that a loss of lung perfusion regulation may account for gas impairment [6]. In our series respiratory system compliance (Crs) was obtained in 18 out of 21 patients who received IMV. Median Crs was 39 ml/cm H2O [IQR: 38–52] and 6 out of 18 patients had a preserved Crs (52 ml/cm H2O [IQR: 52–55]). This subgroup of 6 patients had similar Ang II plasma levels despite renin concentrations significantly higher than the 12 patients with reduced Crs (median renin [IQR] 166.8 pg/ml [114.2–255.2] vs 13.6 [9–66.6], *p* = 0.02). Thus, the development of hypoxemia without marked loss of aerated lung could be explained by the worsening of ventilation perfusion mismatch induced by inadequate Ang II production. These preliminary data suggest that a more efficient RAS axis correlate with a better outcome in patients with ARDS due to SARS-Cov-2. Although the obvious limitations of our small, observational study preclude definitive conclusions, these findings could be considered hypothesis generating for future researches.

**Table 1:** Clinical characteristics, RAS peptides levels and outcomes in the total cohort

Variables	All (*n* = 32)	Survivors (*n* = 18)	Non-Survivors (*n* = 14)	*P* Value
Age, years, median (IQR)	73 (64–78)	69 (56–74)	78 (74–80)	0.001
Males *N* (%)	27 (84)	15 (84)	12 (86)	0.85
SAPS II score, median (IQR)	36 (23–42)	28 (19–36)	40 (31–56)	0.017
SOFA score at ICU admission, median (IQR)	3 (2–6)	3 (2–4)	4 (3–8)	0.115
PaO2/FiO2 ratio at study inclusion, median (IQR)	150 (127–179)	169 (132–181)	143 (113–153)	0.11
Noradrenaline at study inclusion, *N* (%)	12 (37.5)	4 (22)	8 (57)	0.07
**Comorbidities, N (%)**				
Hypertension	16 (50)	9(50)	7 (50)	1
Cardiovascular disease	9 (28)	4 (22)	5 (36)	0.4
Chronic treatment with ACEi and/or ARBs, *N* (%)	11 (34.3)	4 (22.2)	7 (50)	0.25
ACEi and/or ARBs at study inclusion *N* (%)^**(‡)**^	4 (12.5)	2 (11.1)	2 (14.2)	1
**ARDS **^**(†)**^** categories at study inclusion N (%)**				
Mild	9 (28)	5 (28)	4 (29)	1
Moderate	23 (72)	13 (72)	10 (71)	
**Respiratory support throughout the ICU stay N (%)**				
NIV/HFNC	11 (34)	11(61)	0 (0)	< 0.0004
IMV	21 (66)	7 (39)	14 (100)	
AKI ^(^^††)^ at study inclusion, *N* (%)	5 (15.6)	1 (5.5)	4 (28.5)	0.13
ICU LoS, days, median (IQR)	8.5 (5–17)	8 (4–13)	10.5 (7–18)	0.189
**RAS peptides levels at study inclusion **^(*)^				
Renin, pg/mL median (IQR)	16.5 (6.27–105)	13 (4.47–31.77)	56.7 (11–255)	0.04
Angiotensin I, pg/mL, median (IQR)	655 (445–1175)	685 (580–1500)	475 (410–690)	0.03
Angiotensin II, pg/mL, median (IQR)	350 (230–650)	470 (300–920)	250 (220–350)	0.01
Angiotensin 1–7, pg/mL, median (IQR) (**)	230 (170–350)	320 (210–440)	210 (160–250)	0.023
Angiotensin I/Angiotensin II ratio, median (IQR)	1.8 (1.3–2.4)	1.6 (1.3–2.0)	1.9 (1.2–2.9)	0.42
Angiotensin II/ Angiotensin 1–7 ratio, median (IQR)	1.6 (0.96–1.92)	1.66 (0.97–2)	1.58 (0.71–1.72)	0.39

SAPS II, Simplified Acute Physiologic Score Two; SOFA, Sequential Organ Failure Assessment; PaO_2_, arterial partial pressure of oxygen; FiO_2_, Fraction of inspired oxygen; ICU, intensive care unit; ACEi, angiotensin converting enzyme inhibitor; ARBs, angiotensin II receptor blockers; ARDS, acute respiratory distress syndrome; HFNC, high-flow nasal cannula oxygen therapy; NIV, noninvasive mechanical ventilation; IMV, invasive mechanical ventilation; LoS, length of stay; AKI, Acute kidney injury; RAS, renin–angiotensin system; IQR, interquartile range; N, number of patients

^**(‡)**^Seven out of 11 patients who were on chronic treatment with ACEi/ARBs discontinued these medications during hospitalization. The median time from withdrawal of these agents to study inclusion was 6 days [IQR: 4–7]

^**†**^Acute respiratory distress syndrome was defined according to Berlin criteria (2012)

^**††**^Acute kidney injury was defined according to KDIGO criteria (2012)

^*^healthy reference ranges: Renin 1.5–20 pg/ml; Angiotensin I (median 42 pg/ml [IQR:30.46–87.34]); Angiotensin II 25.1 ± 5.2 pg/ml; Angiotensin (1–7) 21.0 ± 4.1 pg/ml

Direct plasma renin levels were determined by chemiluminescent immunoassay. Angiotensin compounds were measured by ELISA assay (Fine Biotech Co., Ltd)

^**^Angiotensin 1–7 plasmatic levels were lower in patients who received IMV compared with those who never received IMV (214 pg/ml [IQR: 163–298] and 335 pg/ml [IQR: 259–499], respectively, *p* = 0.01)

*P* values were calculated by Mann–Whitney test, Chi-square test and Fisher exact test, as appropriate, with a significance level of .05. All statistical analyses were performed using SAS 9.4 software (SAS Institute Inc, Cary, NC, USA)

**Table 2:** RAS peptides levels based on sensitivity analysis ^(*)^

RAS peptides levels at study inclusion ^(**)^	All (*n* = 28)	Survivors (*n* = 16)	Non-survivors (*n* = 12)	*P* value
Renin, pg/mL median (IQR)	15.3 (6.1–79)	13 (4.5–21.3)	77.8 (10.5–256.4)	0.04
Angiotensin I, pg/mL, median (IQR)	579 (408–1156)	996 (579–1510)	439 (400–541)	0.03
Angiotensin II, pg/mL, median (IQR)	276 (217–752)	584 (240–915)	248 (209–301)	0.04
Angiotensin 1–7, pg/mL, median (IQR)	221 (174–355)	328 (214–510)	212 (168–231)	0.04
Angiotensin I/Angiotensin II ratio, median (IQR)	1.8 (1.2–2.6)	1.5 (1.2–2.8)	1.9 (1.3–2.4)	0.86
Angiotensin II/ Angiotensin 1–7 ratio, median (IQR)	1.53 (0.8–2)	1.7 (0.9–2)	1.4 (0.6–1.6)	0.2

^*^We performed a sensitivity analysis excluding 4 patients who were taking ACEi/ARBs at study inclusion

^**^Healthy reference ranges: renin: 1.5–20 pg/ml; Angiotensin I: median 42 [IQR: 30.5–87.3] pg/ml; Angiotensin II: 25.1 ± 5.2 pg/ml; Angiotensin (1–7): 21.0 ± 4.1 pg/ml. Direct plasma renin levels were determined by chemiluminescent immunoassay. Angiotensin compounds were measured by ELISA assay (Fine Biotech Co., Ltd)

*P* values were calculated by Mann–Whitney test, Chi-square test and Fisher exact test, as appropriate, with a significance level of 0.05. All statistical analyses were performed using SAS 9.4 software (SAS Institute Inc, Cary, NC, USA)

## Data Availability

After publication, the data will be made available to others on reasonable requests to the corresponding author. A proposal with detailed description of study objectives and statistical analysis plan will be needed for evaluation of the reasonability of requests. De-identified participant data will be provided after approval from the corresponding author. .
